# Hydroxysafflor Yellow A Shows Protection against PPAR*γ* Inactivation in Nitrosative Neurons

**DOI:** 10.1155/2018/9101740

**Published:** 2018-10-16

**Authors:** Li Sun, Yan-Wei Xu, Jing Han, Chen Xiao, Shan-Shan Cao, Hao Liang, Yan Cheng

**Affiliations:** Tianjin Medical University General Hospital, Tianjin Neurological Institute, Key Laboratory of Post-trauma Neuro-repair and Regeneration in Central Nervous System, Ministry of Education and Tianjin City, Tianjin 300052, China

## Abstract

Peroxynitrite-mediated nitrosative stress in the brain has been associated with various neurodegenerative disorders. Recent evidence highlights peroxisome proliferator-activated receptor *γ* (PPAR*γ*) as a critical neuroprotective factor in neurodegenerative diseases. Here, we observed the effect of the herb hydroxysafflor yellow A (HSYA) during nitrosative stress in neurons and investigated the mechanism based on PPAR*γ* protection. We found that a single exposure of primary neurons to peroxynitrite donor SIN-1 caused neuronal injury, which was accompanied by the increase of PPAR*γ* nitration status and lack of activation of the receptor, as measured by PPAR*γ* DNA-binding activity, by agonist (15d-PGJ2 or rosiglitazone) stimulation. The crucial role of PPAR*γ* in neuronal defense against nitrosative stress was verified by showing that pretreatment with 15d-PGJ2 or rosiglitazone attenuated SIN-1-induced neuronal injury but pretreatment with GW9662, a PPAR*γ* antagonist, aggravated SIN-1-induced neuronal injury. The addition of HSYA not only inhibited SIN-1-induced neuronal damage but prevented PPAR*γ* nitrative modification and resumed PPAR*γ* activity stimulated by either 15d-PGJ2 or rosiglitazone. Furthermore, HSYA also showed the ability to rescue the neuroprotective effect of 15d-PGJ2 or rosiglitazone when the agonists were coincubated with SIN-1. Finally, in vivo experiments demonstrated that the administration of HSYA also efficiently blocked PPAR*γ* nitration and loss of activity in the SIN-1-injected hippocampus and reversed the increased neuronal susceptibility which was supported by the inhibition of Bcl-2 protein downregulation induced by SIN-1. The results suggest that HSYA protects neurons from nitrosative stress through keeping PPAR*γ* as a functional receptor, allowing a more effective activation of this neuroprotective factor by the endogenous or exogenous agonist. Our findings provide new clues in understanding the role of the neuroprotective potential of the herbal HSYA.

## 1. Introduction

Excessively produced nitric oxide (NO) and superoxide lead to the generation of peroxynitrite (ONOO^−^). Peroxynitrite-mediated nitrosative stress causes severe damage to proteins, lipids, and DNA, resulting in cell apoptosis or death. 3-Nitrotyrosine (3-NT) formation has been used extensively as a footprint for the nitrosative stress induced by peroxynitrite [1]. The concentration of 3-NT has been reported to increase in a wide range of neurodegenerative diseases, such as Parkinson's disease, Alzheimer's disease, and traumatic or ischemic brain injury [2–5]. In the ischemic brain, the formation of 3-NT was elevated markedly and the significantly elevated 3-NT was positively correlated with infarct volume in ischemic animals [2]. Also, 3-NT accumulation has been proven to associate with cognitive decline in the AD brain [5]. Furthermore, the inhibition of 3-NT formation protects against brain injury in these disorders [2–5]. Thus, peroxynitrite-mediated nitrosative stress represents an important pathogenic mechanism of neurodegenerative diseases.

Peroxisome proliferator-activated receptor *γ* (PPAR*γ*) is a ligand-activated transcription factor that regulates lipid metabolism and glucose homoeostasis. 15-Deoxy-delta prostaglandin J2 (15d-PGJ2), unsaturated fatty acids, and oxidized phospholipids are PPAR*γ* natural ligands. Its synthetic ligands include the thiazolidinedione (TZD) class of insulin-sensitizing agents (troglitazone, pioglitazone, ciglitazone, and rosiglitazone) and a few of nonsteroidal anti-inflammatory drugs (NSAIDs). Recent studies have shown that, in addition to its classical role, PPAR*γ* activation is neuroprotective against inflammatory reaction and oxidative stress in models of neurodegenerative conditions [6–8]. For example, PPAR*γ* agonist troglitazone or pioglitazone reduced inflammation and infarct volume and improved neurological function following middle cerebral artery occlusion in rats [7]. In cultured hippocampal neurons, rosiglitazone was of protection against mitochondrial damage, oxidative stress, and apoptosis induced by *β*-amyloid (A*β*) [8].

**Figure 1:**
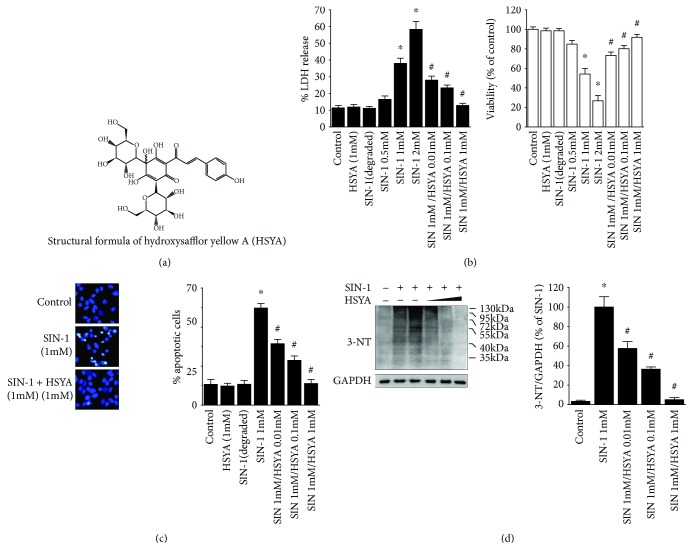
Protective effects of HSYA against SIN-1-induced cytotoxicity in primary neuron cultures. (a) Structural formula of hydroxysafflor yellow A (HSYA). (b–d) The primary neurons were incubated with SIN-1, HSYA, or their combinations as described in Materials and Methods. LDH release assay for cytotoxicity and MTT assay for cell viability (b), Hoechst staining for apoptotic cells (c), and Western blotting for 3-nitrotyrosine (3-NT) expression (d) were carried out after 24 h incubation. Data are expressed as mean ± SEM (*n* = 6). ^∗^*P* < 0.05 compared to control (untreated) and ^#^*P* < 0.05 compared to SIN-1 alone.

Hydroxysafflor yellow A (HSYA) (C27H32O16, MW 612.53), as presented in Figure 1(a), is a water-soluble monomer extracted from the safflower plant (*Carthamus tinctorius* L.). HSYA has been reported to be a natural antioxidant used in traditional Chinese medicine. The antioxidant properties of HSYA in the brain are of particular interest because of the fundamental role that oxidative damage plays in numerous forms of brain diseases. It has been reported that HSYA is able to provide neuroprotective effects via decreasing the level of lipid peroxidation products [9, 10] and inhibiting ROS generation [11]. Recently, HSYA was also demonstrated to modulate endogenous antioxidant defenses of the brain by increasing the activity of antioxidant enzymes, including superoxide dismutase (SOD) and catalase (CAT), as well as the ratio of glutathione (GSH)/glutathione disulfide (GSSG) [12]. However, few studies have investigated the action of HSYA on nitrosative stress of neurons and the underlying mechanism. In this study, we hypothesize that HSYA rescues neurons from nitrosative injury through inhibition PPAR*γ* nitrative modification and inactivation.

## 2. Materials and Methods

### 2.1. Chemicals and Reagents

HSYA was generously provided by Zhejiang Yongning Pharmaceutical Co. Ltd. (Zhejiang, China). The purity of HSYA was >98% determined by HPLC. 3-Morpholino-sydnonimine (SIN-1), 15d-PGJ2, rosiglitazone, GW9662, and mouse antibody to 3-nitrotyrosine were from Cayman Chemical Company (Ann Arbor, MI). Hoechst 33825 and rabbit antibody for 3-nitrotyrosine were purchased from Sigma Chemical Co. (St. Louis, MO). Rabbit antibodies for NeuN and PPAR*γ* and VeriBlot for IP secondary antibody HRP were from Abcam (Cambridge, MA). Mouse antibodies for PPAR*γ* and Bcl-2 were obtained from Santa Cruz Biotechnology (Santa Cruz, CA). All other chemicals were of the highest analytical grades commercially available.

### 2.2. Primary Rat Neurons and Treatments

Primary neurons were prepared from embryonic day 17 Sprague-Dawley rats as previously described [8, 13, 14]. Briefly, cells were dissociated from the hippocampus and maintained in serum-free, B27 neurobasal media (Invitrogen) on poly-D-lysine-coated dishes. After 1 d in vitro, the medium was changed to MEM (Invitrogen) supplemented with 5.5 g/ml D-glucose, 2 mM glutamine, 10% fetal bovine serum (FBS) (Invitrogen), 1 mM sodium pyruvate, 100 U/ml penicillin, and 0.1 mg/ml streptomycin. This medium change was required to reduce excessive antioxidant levels from the B27 medium [14, 15]. Cultures were maintained at 37°C in a 5% CO_2_/95% room air, humidified incubator. On day 3 of culture, cells were treated for 48 h with 0.5 *μ*M cytosine arabinoside to prevent glial growth. On day 9 in culture, the cells formed extensive axonal and dendritic networks and were ready for the experiments. Neuron purity was determined using MAP2 labeling, a cell marker for neurons, which showed >95% purity in cultures.

To expose the cells to various agents, culture medium was replaced by MEM supplemented with 5.5 mg/l D-glucose, 2 mM glutamine, 5% FBS, 100 U/ml penicillin, and 0.1 mg/ml streptomycin. In some studies, cells were incubated with increasing concentrations of SIN-1 (0.05–2 mM, in PBS) for 24 h. In a different set of experiments, cells were exposed to HSYA (0.01–1 mM, in PBS) 10 min prior to the addition of SIN-1 (1 mM) and then coincubated for 24 h. Both SIN-1 and HSYA were prepared immediately prior to use. In an additional set of experiments, the effects of PPAR*γ* agonist and antagonist were observed. To test the effect of PPAR*γ* agonist on PPAR*γ* activation, neurons were incubated with SIN-1 (1 mM) alone or in combination with HSYA (1 mM) for 24 h and then treated with 15d-PGJ2 (5 *μ*M, in PBS) or rosiglitazone (1 *μ*M, in DMSO) for 6 h. To test the effect of PPAR*γ* agonist or antagonist by pretreatment regimen, cells were pretreated for 24 h with the PPAR*γ* agonist (5 *μ*M 15d-PGJ2 or 1 *μ*M rosiglitazone) or PPAR*γ* antagonist (5 *μ*M GW9662, in DMSO) and then exposed to SIN-1 (1 mM) for further 24 h. In the experiments with both PPAR*γ* agonist and PPAR*γ* antagonist, GW9662 was added to the media 10 min prior to PPAR*γ* agonist. To test the effect of PPAR*γ* agonist by cotreatment regimen, cells were exposed to PPAR*γ* agonist with or without HSYA (0.1 mM), 10 min prior to the addition of SIN-1 (1 mM), and then coincubated for 24 h. The concentration of 15d-PGJ2, rosiglitazone, and GW9662 is based on our preliminary concentration-response experiments and the previously published data [8, 16]. In each study, the experimental conditions contained identical concentrations of DMSO which never exceeded 0.1%.

### 2.3. Determination of Lactate Dehydrogenase Activity

Cytotoxicity was quantified by measuring the percentage of total lactate dehydrogenase (LDH) release from cells into the media using the LDH Cytotoxicity Assay Kit (Cayman Chemical, Ann Arbor, MI) following the manufacturers' instructions. Cells were treated with SIN-1 alone or in various combinations with other agents. 24 h after the initiation of SIN-1 treatment, the supernatant (100 *μ*l) was transferred to a 96-well plate for the measurement of LDH activity. The percentage of LDH released into the media was calculated by the following formula: (LDH activity in the media/total LDH activity) × 100, where total LDH activity represents LDH activity in cells and media. Total LDH was determined in cells treated with 0.1% Triton X-100.

### 2.4. Cell Viability Assay

To assess neuronal viability, 3-(4,5-dimethylthiazol-2-yl)-2,5-diphenyl-tetrazolium bromide (MTT) assay was performed. The principle of the assay is based on the cleavage of tetrazolium salts by mitochondrial succinate reductase in viable cells to form formazan dye. Briefly, MTT solution (0.5 mg/ml) was added to the culture well 24 h after SIN-1 treatment. Following incubation for 4 h at 37°C, the formed formazan crystals were dissolved in DMSO. The absorbance of each well was measured at 570 nm using an automatic plate reader, and the cell viability was expressed as percent of control.

### 2.5. Hoechst 33258 Staining

Cell apoptosis was measured by the procedure described previously using Hoechst 33258 stain [8, 17]. Changes in nuclear morphology characteristics of apoptosis were observed in cells labeled with Hoechst 33258. The nuclei in normal cells presented uniformly hypochromatic blue color, and the nuclei in apoptotic cells presented fragmented and condensed staining. The number of apoptotic nuclei in at least 10 randomly chosen fields was counted and expressed as percent of total cells.

The cell loss in the hippocampus of rats was measured by counting the numbers of the cell nuclei stained with Hoechst 33258. Six preselected areas of the hippocampus were counted per each animal. Six animals from each group were used for the analyses. Each area subjected to the cell nuclei counting was set as 300 *μ*m × 300 *μ*m.

### 2.6. Western Blot Analysis

Samples were separated by SDS-PAGE and then transferred onto the nitrocellulose membrane. After blocking for 1 h in 0.1% Tween 20/PBS containing 5% fat-free milk, the blot was then incubated with anti-PPAR*γ* antibody, anti-nitrotyrosine antibody, or anti-Bcl-2 antibody at 4°C overnight. After incubation with the appropriate HRP-conjugated secondary antibodies, the blot was visualized by chemiluminescence. The density of the bands was evaluated densitometrically using the program Quantity One 4.6.2 (Bio-Rad Laboratories, Hercules, CA). The specificity of the bands for nitrated tyrosine was confirmed in pilot experiments of Western blot. The SDS-PAGE-transferred membrane was incubated with anti-nitrotyrosine antibody that was preabsorpted for 4 h with an excess of free nitrotyrosine (10 mM), aminotyrosine (10 mM), phosphotyrosine (10 mM), methyltyrosine (10 mM), or tyrosine (10 mM). The nitrated protein bands were abolished by preabsorption of the antibody with nitrotyrosine but not aminotyrosine, phosphotyrosine, methyltyrosine, or tyrosine. This method is also used by others to verify the specificity of the assay for protein tyrosine nitration [18].

### 2.7. Immunoprecipitation Analysis

For immunoprecipitation assay, samples were precleared with protein A/G agarose bead slurry on a shaker at 4°C for 10 min to remove the nonspecific binding protein. The protein A/G beads were removed by spin at 14,000 g at 4°C for 10 min. The supernatant (500 *μ*g protein in 0.5 mg/ml) was incubated with 2 *μ*g mouse anti-PPAR*γ* or anti-IgG (control) antibodies and rotated at 4°C for 3 h. The Ag/Ab immunocomplexes were captured by adding protein A/G agarose beads and rocked at 4°C overnight. Agarose beads were collected by centrifugation at 14,000 g at 4°C for 10 min and then washed three times in PBS. Finally, immunocomplexes were dissociated from agarose beads by boiling with SDS-PAGE sample buffer for 5 min and Western blotting was performed with rabbit anti-3-nitrotyrosine antibody to detect the nitrated PPAR*γ*. A HRP-conjugated VeriBlot for IP detection reagent was used to exclude interference from the antibody heavy and light chains.

### 2.8. PPAR*γ* DNA-Binding Assay

PPAR*γ* activity was quantified by PPAR*γ* DNA-binding assay using a sensitive and specific TransAM PPAR*γ* transcription factor assay kit (Active Motif, Carlsbad, CA, USA), as we described [13]. This assay measures the capacity of PPAR*γ* binding to an oligonucleotide probe that contains the specific peroxisome proliferator response element (PPRE), immobilized on a 96-well plate. Nuclear proteins were isolated with a nuclear protein extraction kit (Active Motif) at 6 h following the initiation of the treatment with PPAR*γ* agonist. 10 *μ*g of nuclear extract protein was applied to the wells and allowed to bind to the PPRE. Bound PPAR*γ* was then detected by adding the specific anti-PPAR*γ* primary antibody, an HRP-conjugated secondary antibody, and HRP substrate solution and spectrophotometer reading (450 nm). The specificity of the assay was confirmed by the addition of wild-type and mutated consensus oligonucleotides. The wild-type consensus oligonucleotide can prevent PPAR*γ* binding to the probe, whereas the mutated consensus oligonucleotide has little effect on PPAR*γ* binding.

### 2.9. Immunohistochemistry

Fresh-frozen sections were stained for 3-NT as we described [13]. Briefly, sections were permeabilized with 0.3% (*v*/*v*) Triton X-100 in PBS for 30 min and blocked with 1% (*w*/*v*) BSA in PBS for 1 h and then incubated with mouse monoclonal antibody for 3-NT (1 : 200) at 4°C overnight. After rinsing with PBS, sections were incubated with rhodamine isothiocyanate (TRITC) labeled goat anti-mouse antibody for 1 hr at 37°C in the dark. The fluorescent images were observed under a fluorescent microscope. For double labeling, sections were incubated first with antibody of mouse anti-nitrotyrosine (1 : 200) followed by a specific neuron marker antibody of rabbit anti-NeuN (1 : 200) or an antibody of rabbit anti-PPAR*γ* (1 : 200). Following three washes in PBS, immune complexes were visualized with Texas Red conjugated anti-mouse IgG (1 : 500) and FITC conjugated anti-rabbit IgG (1 : 500). The specificity of staining was confirmed by replacement of the primary antibody with nonimmune control IgG or by elimination of the primary antibody.

### 2.10. Hippocampus Injection and Treatments

All animal experiments were carried out according to an institutionally approved protocol, in accordance with the National Institutes of Health Guide for the Care and Use of Laboratory Animals, and were approved by the Institutional Animal Care and Use Committee of Tianjin Medical University. Male Sprague-Dawley (SD) rats (Academy of Military Medical Sciences, Beijing, China) weighing from 280 to 330 g were housed and cared for in the Animal Resource Center under 12 h light-dark cycles and allowed free access to food and water. All animal manipulations were conducted during the lights-on phase (0700–1900 h). Briefly, anesthetized rats were placed in a stereotaxic apparatus and 3 *μ*l SIN-1 (25 mM) in PBS was infused into the right hippocampus using the following coordinates: 4.0 mm posterior to the bregma, 2.0 mm lateral from midline, and 4.0 mm below the dural surface. The above procedures were completed under sterile conditions, and penicillin (200,000 U, intramuscularly) was injected to prevent infection. The dosage of SIN-1 was chosen according to the previously published study, in which the dose 25 mM was found to be the most effective in inducing protein nitration by hippocampus injection [17]. The control group was injected with the same volume of vehicle. Body temperature was maintained at 37°C with the use of a heating pad throughout the surgery procedure and until animals regained consciousness. Thereafter, animals were returned to their home cages and allowed free access to food and water.

HSYA dissolved in PBS was administered intravenously through the caudal vein at a dose of 1, 5, or 10 mg/kg 30 min before SIN-1 treatment. Our previous experiments have shown the neuroprotective effects of HSYA injected within this range of dosage in ischemia/reperfusion rats [2]. The ability of HSYA to cross the blood-brain barrier (BBB) following intravenous administration has been confirmed previously [19]. At 24 h after hippocampal injection with SIN-1, 10 *μ*g of 15d-PGJ2 in 10 *μ*l of PBS was administered intracerebroventricularly (ICV) at a rate of 1 *μ*l/min using a syringe pump as described in our previous study [13]. The effect of vehicle without any drug was tested in pilot experiments, and no effects were observed.

### 2.11. Statistical Analysis

The experimental data are expressed as mean ± SEM, and SPSS 11.0 software package was used for data processing. One-way ANOVA was used to compare the means of different groups. Comparisons between two groups were conducted by *t*-test. A *P* value less than 0.05 was considered as statistically significant.

## 3. Results

### 3.1. HSYA Protected Neurons from SIN-1-Induced Cytotoxicity

To model nitrosative damage, primary hippocampal neurons (d 9) were exposed to SIN-1, a well-known peroxynitrite donor, for 24 h. As expected, neurons treated with SIN-1 exhibited cytotoxic damage, as determined by LDH released into the media and MTT assay for cell viability (Figure 1(b)), the Hoechst 33258 staining assay for apoptotic nuclei (Figure 1(c)), or protein nitrative modification based on 3-NT formation (Figure 1(d)).

To observe the effect of HSYA on SIN-1-induced cytotoxicity, varying concentrations of HSYA were added to the media together with a toxic level of SIN-1. As illustrated in Figure 1(b), inclusion of HSYA resulted in decreases in LDH release induced by SIN-1. Similar conclusions demonstrating a neuroprotective effect of HSYA were generated through measurement of MTT assay (Figure 1(b)), the Hoechst 33258 staining (Figure 1(c)), and 3-NT accumulation (Figure 1(d)).

### 3.2. HSYA Inhibited SIN-1-Induced PPAR*γ* Nitration and Inactivation

**Figure 2:**
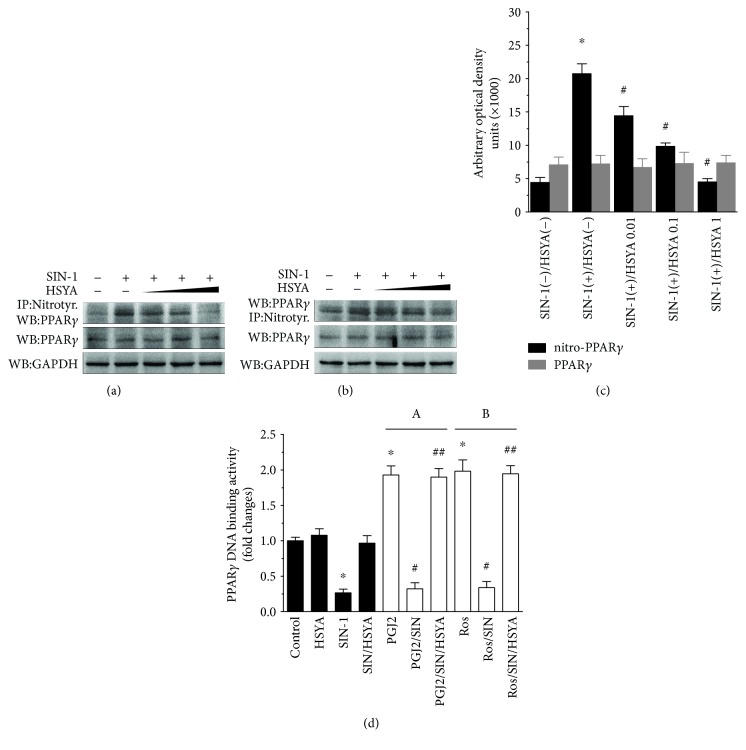
Protection against SIN-1-induced PPAR*γ* nitration and inactivation by HSYA in primary neurons. (a–c) Inhibition of PPAR*γ* nitration by HSYA in SIN-1-treated neurons. The neurons were incubated with increasing concentrations of HSYA (0.01, 0.1, and 1 mM), 10 min before the addition of SIN-1 (1 mM). After 24 h coincubation, neurons were harvested for analysis of PPAR*γ* nitration and total PPAR*γ* accumulation. GAPDH expression was shown as a loading control. (a) The cell extracts were immunoprecipitated (IP) with antibody specific to nitrotyrosine (Nitrotyr.). The nitrotyrosine immunoprecipitates were successively immunoblotted (WB) with PPAR*γ* Ab. (b) The cell extracts were IP with anti-PPAR*γ* antibody followed by WB with nitrotyrosine Ab. The bar graph illustrates the densitometric analysis of the related bands. Data are expressed as mean ± SEM (*n* = 3). ^∗^*P* < 0.05 compared to control (untreated) and ^#^*P* < 0.05 compared to SIN-1 alone. (d) Restoration of agonist-dependent PPAR*γ* activation by HSYA in SIN-1-treated neurons. Primary neuron cultures were incubated with 1 mM HSYA, 10 min before the addition of 1 mM SIN-1. After 24 h coincubation, cells were treated for an additional 6 h in the absence (filled bars) or presence (open bars) of PPAR*γ* agonist 15d-PGJ2 (5 *μ*M) A or rosiglitazone (Ros) (1 *μ*M) B. Nuclear proteins were extracted, and activated PPAR*γ* was quantified by PPAR*γ* DNA-binding activity utilizing the PPAR*γ* transcription factor assay kit. Data are expressed as mean ± SEM (*n* = 3). ^∗^*P* < 0.05 compared to control (untreated), ^#^*P* < 0.05 compared to PPAR*γ* agonist (15d-PGJ2 or Ros) alone, and ^##^*P* < 0.05 compared to SIN-1 plus agonist (15d-PGJ2 or Ros).

Searching for a possible mechanism to explain the beneficial effect of HSYA, we considered PPAR*γ* which is an important factor in the neuronal defense mechanisms against oxidative injuries [8, 20]. PPAR*γ* has been described to be modified and inactivated by nitration of tyrosine residues in nonneuronal cells [21]. To determine neuronal PPAR*γ* sensitivity to nitration, the level of PPAR*γ* in the nitrated form was detected at 24 h following the exposure to SIN-1 with or without HSYA. The presence of nitrated PPAR*γ* (nitro-PPAR*γ*) was examined by immunoprecipitating proteins from cellular extract with anti-PPAR*γ* antibody, and then the PPAR*γ* immunoprecipitates were immunoblotted with anti-3-NT antibody (Figure 2(a)). Also, the reciprocal experiment was carried out by immunoprecipitating proteins with 3-NT antibody first and then immunoblotting with PPAR*γ* antibody (Figure 2(b)). In the results of both experiments, SIN-1 treatment resulted in an increase of PPAR*γ* nitration, which was reversed by the cotreatment of HSYA in a concentration-dependent manner. However, the abundance of PPAR*γ* protein was not affected by either SIN-1 alone or in combination with HSYA.

Nitrative modification could leave PPAR*γ* to become refractory to the activation by its activating agents [21]. We then evaluated whether SIN-1 exposure affected the response of PPAR*γ* to its ligand stimulation. The neurons in culture were incubated with 15d-PGJ2, a natural ligand for PPAR*γ*, for 6 h following the 24 h exposure to SIN-1 alone or in combination with HSYA. PPAR*γ* DNA-binding activity was increased about 2-fold by the exposure to 15d-PGJ2 alone, indicating the activation of PPAR*γ* (Figure 2(d), A). SIN-1 treatment inhibited 15d-PGJ2-induced elevation in PPAR*γ* DNA-binding activity, which was restored by the presence of HSYA (Figure 2(d), A). In analogy to results with 15d-PGJ2, HSYA also resumed PPAR*γ* activation by rosiglitazone, a synthetic agonist for PPAR*γ*, in SIN-1-treated neuron cultures (Figure 2(d), B). Notably, a significant reduction in PPAR*γ* activity was detected following SIN-1 exposure alone, suggesting the loss of PPAR*γ* response to endogenous ligands, whereas treatment of HSYA with SIN-1 fully compensated for this SIN-1-induced dysfunction (Figure 2(d), filled bars). Overall, the HSYA-mediated protection of PPAR*γ* activity was consistent with improved neuronal damage.

Finally, the effect of HSYA on PPAR*γ* was also observed in normal neurons. No significant alterations in either PPAR*γ* protein expression or DNA-binding activity were detected (data not shown), suggesting that HSYA itself did not emerged as a direct inducer of PPAR*γ* activity.

### 3.3. HSYA Resumed the Protective Effect of PPAR*γ* Agonists against SIN-1-Induced Cytotoxicity

**Figure 3:**
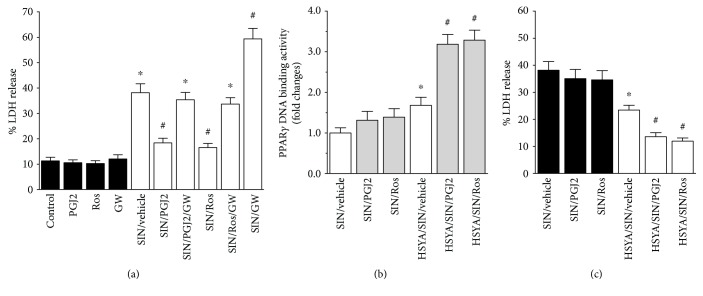
Additional protection against SIN-1-induced neuronal injury by HSYA combined with PPAR*γ* agonist. (a) PPAR*γ*-mediated protection of neurons from SIN-1-induced cytotoxicity. The neurons were pretreated for 24 h with the PPAR*γ* agonist 15d-PGJ2 (PGJ2) (5 *μ*M) or rosiglitazone (Ros) (1 *μ*M), antagonist GW9662 (GW) (5 *μ*M), or their combination as described in Materials and methods. Cultures were then incubated with or without 1 mM SIN-1 (SIN) for further 24 h. LDH release to the media was employed as neuronal damage index. Data are presented as mean ± SEM (*n* = 3). ^∗^*P* < 0.05 compared to non-SIN-1-treated groups and ^#^*P* < 0.05 compared to SIN-1 alone. (b, c) Additional protection against SIN-1-induced neuronal injury by HSYA combined with PPAR*γ* agonist. The PPAR*γ* agonist (5 *μ*M 15d-PGJ2 or 1 *μ*M rosiglitazone) with or without HSYA (0.1 mM) was added to the cultures 10 min prior to SIN-1 (1 mM) exposure. Activated PPAR*γ* (b) and neuronal insult (c) were evaluated by PPAR*γ* DNA-binding activity at 6 h and LDH release assay at 24 h, respectively, after the coincubation with SIN-1. Data are presented as mean ± SEM (*n* = 3). ^∗^*P* < 0.05 compared to SIN-1 alone and ^#^*P* < 0.05 compared to SIN-1 plus HSYA alone.

To determine whether PPAR*γ* activity plays a crucial role in the defense against SIN-1-induced nitrosative stress, the PPAR*γ*-specific agonist and/or antagonist was added to the cultures 24 h prior to the treatment with a toxic level of SIN-1. As demonstrated in Figure 3(a), PPAR*γ* agonist (15d-PGJ2 or rosiglitazone) pretreatment significantly attenuated SIN-1-induced LDH release, which was reversed by the copretreatment of PPAR*γ* antagonist GW9662. Alternatively, pretreatment with GW9662 alone aggravated SIN-1-induced neuronal injury (Figure 3(a)). These results suggested that PPAR*γ* activation could increase resistance to SIN-1 cytotoxicity whereas PPAR*γ* inactivation caused neurons to be more sensitive to SIN-1-induced insult.

In another experiment, cotreatment of PPAR*γ* agonist (15d-PGJ2 or rosiglitazone) with SIN-1, however, failed to either activate PPAR*γ* (Figure 3(b)) or protect neurons against SIN-1-induced cytotoxicity (Figure 3(c)), indicating that a preactivation of PPAR*γ* is required to inhibit neuronal insult by SIN-1. Alternatively, the nitration of PPAR*γ* induced by SIN-1 could prevent PPAR*γ* activation and thus the neuroprotection by PPAR*γ* agonist. To verify this last hypothesis, neurons were exposed to HSYA, at a submaximal concentration, together with PPAR*γ* agonist plus SIN-1. As demonstrated in Figures 3(b) and 3(c), the combined treatment of HSYA and PPAR*γ* agonist not only rescued PPAR*γ* response to its activating agents (Figure 3(b)) but afforded additional protection against SIN-1-induced cell insult when compared with the HSYA plus SIN group (Figure 3(c)). These findings suggested that HSYA not only itself has neuroprotective capacity but could help to resume PPAR*γ* agonist-based protection against SIN-1-induced insult.

### 3.4. HSYA Inhibited PPAR*γ* Nitration and Loss of Activity in the SIN-1-Injected Hippocampus of Rats

**Figure 4:**
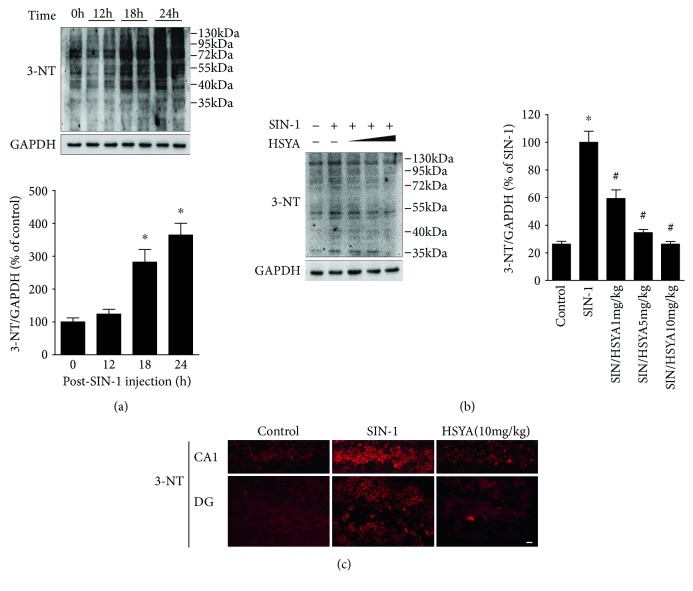
The inhibitory effect of HSYA on 3-nitrotyrosine accumulation in the SIN-1-injected rat hippocampus. (a) The time-dependent induction of 3-nitrotyrosine (3-NT) formation by SIN-1. SIN-1 (25 mM) was injected to the rat hippocampus, and the abundance of 3-NT was measured at 0, 12, 18, and 24 h after the injection by Western blotting. The bar graph illustrates the densitometrical analysis of nitrotyrosine protein mass (normalized by GAPDH). Data are expressed as mean ± SEM (*n* = 3). ^∗^*P* < 0.05 compared to control (untreated). (b, c) Reduced 3-NT accumulation by HSYA in the SIN-1-injected hippocampus. Rats were treated intravenously with increasing doses of HSYA (1, 5, and 10 mg/kg), 30 min before SIN-1 injection. The effect of HSYA on 3-NT accumulation was determined at 24 h after SIN-1 injection by Western blotting (b) and immunofluorescence (c). The bar graph illustrates the densitometrical analysis of nitrotyrosine protein mass. Data are expressed as mean ± SEM (*n* = 3). ^∗^*P* < 0.05 compared to control (untreated) and ^#^*P* < 0.05 compared to SIN-1 alone. Photomicrographs show the labeling of 3-NT (red) in the CA1 and dentate gyrus (DG) areas. Scale bar, 20 *μ*m.

To determine whether HSYA has similar effects on PPAR*γ in vivo*, we employed an animal model of nitrosative stress based on hippocampus injection of SIN-1. To confirm the production of peroxynitrite in the SIN-1-injected hippocampus, 3-NT expression was measured at 24 h following SIN-1 injection.  Figure 4(a) displayed that SIN-1 induced a time-dependent increase in 3-NT abundance, which was more than 3-fold higher than the one observed in control rats, for 24-hour-treated rats. Administration of HSYA significantly ameliorated 3-NT expression and immunoreactivity in the SIN-1-injected hippocampus (Figures 4(b) and 4(c)).

**Figure 5:**
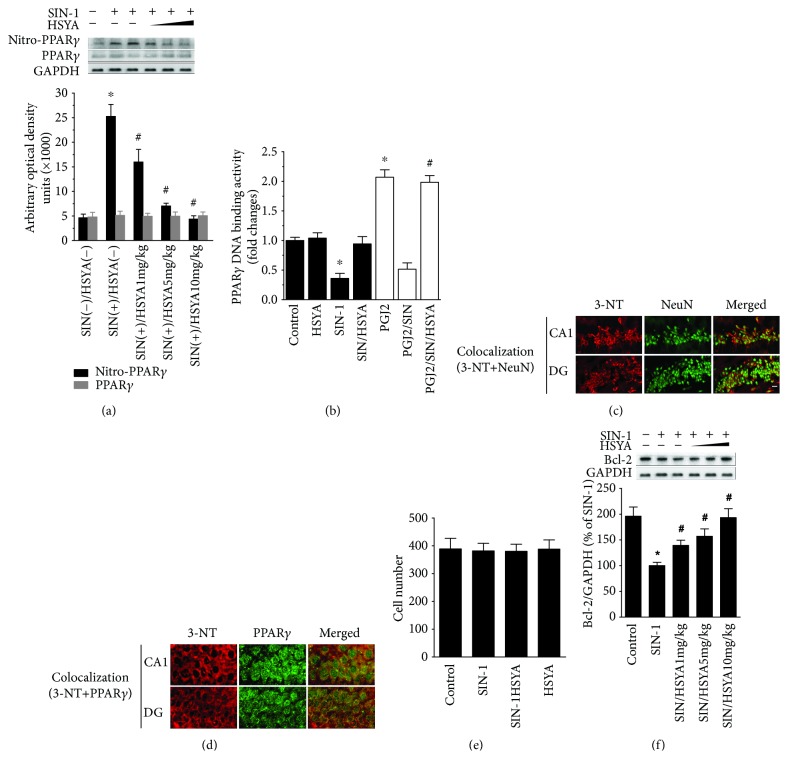
The inhibitory effects of HSYA on PPAR*γ* nitration and inactivation in the SIN-1-injected rat hippocampus. (a) Inhibition of SIN-1-induced PPAR*γ* nitration by HSYA in the hippocampus of rats. Rats were treated with increasing doses of HSYA (1, 5, or 10 mg/kg), 30 min before SIN-1 (25 mM) injection. The effects of HSYA were evaluated at 24 h after SIN-1 injection. The protein levels of nitro-PPAR*γ* and PPAR*γ* were characterized by immunoprecipitation or Western blotting. The bar graphs illustrate the densitometric analysis of the related bands. Data are expressed as mean ± SEM (*n* = 3). ^∗^*P* < 0.05 compared to control (untreated) and ^#^*P* < 0.05 compared to SIN-1 alone. (b) Prevention of PPAR*γ* inactivation by HSYA in the SIN-1-injected hippocampus. Rats were injected with HSYA (10 mg/kg), SIN-1 (25 mM), or their combination as described in Materials and Methods. At 24 h postinjection, rats were treated intracerebroventricularly (ICV) with either vehicle (filled bars) or 10 *μ*g 15d-PGJ2 (open bars). Hippocampal nuclear extracts were prepared for the detection of PPAR*γ* DNA-binding activity 6 h after ICV administration. Data are expressed as mean ± SEM (*n* = 3). ^∗^*P* < 0.05 compared to control (untreated) and ^#^*P* < 0.05 compared to SIN-1 plus 15d-PGJ2. (c, d) Localization of nitrated PPAR*γ* in the SIN-1-injected hippocampus. The rat hippocampus was injected with SIN-1 (25 mM). The neuronal distribution of nitro-PPAR*γ* in the hippocampus was detected by immunofluorescent double labeling at 24 h after SIN-1 injection. (c) Photomicrographs show the colocalization of 3-NT (red) with NeuN (green) in the CA1 and dentate gyrus (DG) areas of the hippocampus. Scale bar, 20 *μ*m. (d) Photomicrographs with increased magnification show the colocalization of PPAR*γ* (green) with 3-NT (red) in the cytoplasm of most 3-NT-positive cells. (e) Rats were treated with HSYA (10 mg/kg), 30 min before SIN-1 (25 mM) injection. At 24 h postinjection, the cell loss was measured by counting the numbers of Hoechst 33258-stained nuclei. Summary bar graph illustrates cell counts in the hippocampus. Data are expressed as mean ± SEM (*n* = 6 fields counted in 6 animals). (f) Inhibition of SIN-1-induced Bcl2 expression by HSYA in the hippocampus of rats. Rats received HSYA treatment of 1, 5, or 10 mg/kg 30 min before SIN-1 (25 mM) injection. The effects of HSYA on the protein levels of Bcl-2, a downstream target of PPAR*γ* signaling, were evaluated by Western blotting at 24 h after SIN-1 injection. The bar graphs illustrate the densitometric analysis of the related bands. Data are expressed as mean ± SEM (*n* = 3). ^∗^*P* < 0.05 compared to control (untreated) and ^#^*P* < 0.05 compared to SIN-1 alone.

We then examined the effect of HSYA on nitro-PPAR*γ* expression and PPAR*γ* activity. As shown in Figure 5(a), HSYA inhibited nitro-PPAR*γ* generation induced by SIN-1 injection in a dose-dependent manner (Figure 5(a)). PPAR*γ* protein expression was not affected by either SIN-1 alone or coinjection with HSYA (Figure 5(a)). Consistently, a reduced PPAR*γ* DNA-binding activity was found in the SIN-1-injected hippocampus, which was reversed by HSYA treatment (Figure 5(b)). HSYA also resumed PPAR*γ* response to its ligand 15d-PGJ2 in the SIN-1-injected hippocampus (Figure 5(b)). The cellular distribution of nitro-PPAR*γ* in the hippocampus was also characterized. As shown in Figures 5(c) and 5(d), the immunoreactivity of 3-NT was found primarily in neurons as indicated by its colocalization with a neuronal cell marker NeuN, suggesting that the induced protein nitration is likely a result of the neuronal response to SIN-1 injection. Concomitantly, the clear overlay of PPAR*γ* signal with 3-NT signal, representing nitro-PPAR*γ*, was observed in the cytoplasm of most 3-NT-positive cells, implicating that nitro-PPAR*γ* also preferentially occurred in neurons of the hippocampus.

In contrast to SIN-1-induced insults in cultured neurons, no obvious cell loss or apoptosis-like morphology was observed in rats injected with SIN-1 alone or coinjected with HSYA, as assessed by Hoechst 33258 staining (Figure 5(e)), indicating that the single injection of SIN-1 was not sufficient to cause cell loss and cell apoptosis. Furthermore, no significant difference in spatial memory retention, a process associated with the hippocampus, could be detected in the Morris water maze test (data not included). These results were consistent with the previous report of an SIN-1-injected hippocampus [17]. PPAR*γ* loss of function in neurons, however, has been proven to be associated with increased susceptibility to oxidative stress, which is reflected in downregulation of the Bcl-2 antiapoptotic protein [8]. Accordingly, Bcl-2 protein expression was determined in the hippocampus. As the changes occurred in PPAR*γ* activity, similar downregulation and upregulation of Bcl-2 protein expression were observed in SIN-1-injected and HSYA-coinjected rats, respectively (Figure 5(f)), suggesting the increased vulnerability to damage in the SIN-1-injected hippocampus and the potential properties of HSYA to decrease this predisposition.

Administration of HSYA alone to normal rats had no significant effect on any of the measured indices (data not included).

## 4. Discussion

Our experiments demonstrated that SIN-1-induced neuronal damage or increased vulnerability was notably reduced by the herb HSYA. This neuroprotective effect was established in both neurons in culture and animal models of nitrosative stress. We further demonstrated that the neuroprotective effect of HSYA may be associated with inhibition of PPAR*γ* nitration and inactivation induced by SIN-1. Next, in support of the above statement, the crucial role of PPAR*γ* in neuronal defense against nitrosative stress was verified by showing the evidence that the PPAR*γ* agonists attenuated SIN-1-induced neuronal injury but the PPAR*γ* antagonist aggravated SIN-1-induced neuronal injury. Finally, we postulated that HSYA may potentiate the PPAR*γ*-mediated neuroprotective effects by inhibition of PPAR*γ* inactivation since the combined treatment of HSYA with PPAR*γ* agonist rescued the effects of agonist on both PPAR*γ* activation and PPAR*γ* protection against SIN-1-induced cytotoxicity.

Evidence has proven that PPAR*γ* is important in neuronal self-defense against oxidative injuries. For example, in PC12 neuronal cell, PPAR*γ* loss of function increased susceptibility to H_2_O_2_- or *β*-amyloid- (A*β-*) induced oxidative toxicity, whereas PPAR*γ* overexpression could prevent H_2_O_2_- or A*β*-induced ROS production and cell insult [8]. Consistently, increased brain damage and oxidative stress were observed in neuronal PPAR*γ* knockout (N-PPAR*γ*-KO) mice in response to middle cerebral artery occlusion [20]. Also, the primary neurons from N-PPAR*γ*-KO mice were significantly more vulnerable to oxidative injury, albeit deficiency of PPAR*γ* did not affect the baseline neuronal health [20]. In support of this notion, our study demonstrated that PPAR*γ* may also contribute to the defensive mechanism against nitrosative stress in neurons by showing that PPAR*γ* agonist attenuated SIN-1-induced cytotoxicity but PPAR*γ* antagonist enhanced SIN-1-induced cytotoxicity. Indeed, in our study, a certain level of PPAR*γ* activity was demonstrated in the control neuron cells and the hippocampus, suggesting the activation of PPAR*γ* by endogenous natural agonists, such as 15d-PGJ2 or oxidized lipids, in the normal settings of the brain. The SIN-1-induced decrease in PPAR*γ* activity in our study indicated the loss of PPAR*γ* response to these endogenous ligands. Consistently, exogenous administration of 15d-PGJ2, whose production has been proved to be increased by SIN-1 [22], was unable to increase PPAR*γ* activity in the SIN-1-treated neurons and hippocampus. Thus, we speculated that SIN-1-induced PPAR*γ* inactivation may dampen the PPAR*γ*-mediated defense system and increase neuronal vulnerability to damage, whereas HSYA's inhibition of PPAR*γ* inactivation may protect the defense system, thus conferring more resistance to nitrosative stress on neurons.

In addition to the endogenously produced agonists, PPAR*γ* also became refractory to the stimulation of exogenously added agonists after inactivation by SIN-1. This could help explaining some findings in PPAR*γ* agonist-based therapy against nitrosative stress, which show that PPAR*γ* agonists have actions without PPAR*γ* activation. It has been shown in a MPP^+^/MPTP model of Parkinson's disease that PPAR*γ* activity was important for protecting against MPTP toxicity. However, only non-PPAR*γ*-mediated neuroprotective effects of rosiglitazone were observed in MPTP-treated mice, as these actions of rosiglitazone were not associated with the upregulation of PPAR*γ* target gene and could not be reversed by cotreatment with PPAR*γ* antagonist [23]. Consistently, in a model of brain trauma, whose pathogenesis also involves nitrosative stress, PPAR*γ* agonist pioglitazone demonstrated beneficial functions through mechanisms not related to PPAR*γ* activation [24]. In agreement with these findings, NCX 2216, the NSAID class of PPAR*γ* agonist, controls microglial activation through PPAR*γ*-dependent and PPAR*γ*-independent actions. Prolonged treatment of microglial cultures with NCX 2216 can induce PPAR*γ* nitration. Following nitration, NCX 2216 can no longer demonstrate the effects associated with PPAR*γ* activation; however, its PPAR*γ*-independent effects were still being observed in microglial cultures [25]. In our study, the PPAR*γ* agonist significantly inhibited SIN-1-induced cytotoxicity in neurons whereas the specific PPAR*γ* antagonist prevented such inhibition, suggesting that the effect was mediated by PPAR*γ*. However, the PPAR*γ*-mediated effect was observed in pretreatment regimen of PPAR*γ* agonist but not in cotreatment regimen. We speculated that the ineffectiveness of cotreatment may be due to PPAR*γ* nitration and subsequent inactivation, since the cotreatment of HSYA in combination with PPAR*γ* agonist demonstrated synergistic effects on PPAR*γ* activation and cytoprotection. Further investigations are required to confirm whether this synergistic action of HSYA is associated with the inhibition of PPAR*γ* nitrative modification, thus allowing a more effective activation of PPAR*γ* by the agonists.

In our *in vivo* study, the SIN-1-induced PPAR*γ* nitration and inactivation were suggested to be mainly occurring in neurons of the hippocampus. Concurring with that of our result, the tau protein has been identified as one of the targets of peroxynitrite and the nitrated tau protein was also suggested to have a strong neuronal signature [17]. Similarly, the peroxynitrite-induced 3-NT expression was also proved to be primarily accumulated in neurons in the acute phase of cerebral ischemia/reperfusion injury [26], albeit no target protein for nitration was evaluated. The reason why proteins in neurons are prone to nitrative modification by peroxynitrite may be related with the lower concentrations of antioxidants in neurons than in glial cells [27]. Of particular note is the low concentration of reduced glutathione (GSH). The susceptibility of cells to peroxynitrite toxicity has been proven to largely depend on the amount of intracellular GSH [28]. The GSH concentration in neurons is one-half of that of astrocytes, and the activity of *γ*-glutamylcysteine synthetase, a key enzyme in glutathione synthesis, is approximately several fold lower in neurons than that of astrocytes. [27]. This low antioxidative potential may be predisposed to neuronal PPAR*γ* nitration and inactivation in response to peroxynitrite injury. On the other hand, neurons rely heavily on their metabolic coupling with astrocytes to combat oxidative stress. Astrocytes produce and secret GSH to protect neurons as well as provide the precursors for neuronal GSH synthesis [14, 29]. Without the antioxidant support from astrocytes, neurons are of high susceptibility to the oxidative damage [14, 29]. This may provide a likely explanation for the observation in our study showing that there is SIN-1-induced significant cell injury in the neuron-enriched culture system whereas no toxicity (apoptosis or cell loss) to cells in the SIN-1-injected hippocampus was observed.

The Bcl-2 antiapoptotic protein has been shown to be a key downstream target of PPAR*γ* signaling in neurons for protection against oxidative stress [8, 30, 31]. PPAR*γ* loss of function results in downregulation of Bcl-2 protein in neurons and thereby renders cell vulnerable to oxidative insult [8]. PPAR*γ* agonists protected neurons against oxidative damage by enhancing Bcl-2 expression [8, 30]. Moreover, a putative PPAR*γ* response element (PPRE) has been reported in the 3′-untranslated region of the *bcl-2* gene [32], suggesting the dependence of PPAR*γ*. Consistently, in our study, PPAR*γ* nitration and inactivation were accompanied by a parallel decrease in Bcl-2 expression in the SIN-1-injected hippocampus. This data may provide an additional link to our speculation that SIN-1-induced PPAR*γ* nitration and inactivation *in vivo* may be reflected in the increased vulnerability to brain damage. Concomitantly, the inhibition of Bcl-2 downregulation induced by SIN-1 supported the protective effect of HSYA. Further study is warranted to confirm the association between PPAR*γ* and Bcl-2 in our study.

In the nervous system, protein tyrosine nitration represents a major cytotoxic pathway during peroxynitrite-mediated nitrosative stress. However, other covalent modifications of PPAR*γ* cannot be excluded to explain some of our observations. For example, PPAR*γ* is known to be modified by phosphorylation at serine residue [33]. Studies have demonstrated that peroxynitrite, acting as a signaling molecule, regulates mitogen-activated protein kinase- (MAPK-) mediated signal transduction pathways. PPAR*γ* contains a MAPK site, and phosphorylation by extracellular signal-regulated kinase- (ERK-) 1/2 leads to inhibition of PPAR*γ* activity [33]. It was reported that peroxynitrite potently activated ERK1/2 in a wide variety of cell types, including neural cells [34, 35]. Thus, PPAR*γ* is possibly susceptible to the modification of phosphorylation in nitrosative conditions. We assumed that increased tyrosine nitration after SIN-1 treatment was the primary reason for the inactivation of PPAR*γ*. However, to be sure of this assumption, the degree of PPAR*γ* nitration without alterations of other amino acids will have to be determined in future studies. Additionally, the specific tyrosine residue in PPAR*γ* that is nitrated is also required to be determined.

Actually, many additional transcription factors exhibit sensitivity to both reactive oxygen species and nitric oxide-related species, e.g., NF-*κ*B, AP-1, and p53. Posttranslational modification plays an important role in regulating the activity of transcription factors during oxidative and nitrosative stresses. For example, studies have demonstrated that NF-*κ*B resides in the cytoplasm in an inactive complex with the inhibitor I*κ*Bs and oxidizing conditions in the cytoplasm promote NF-*κ*B activation. Phosphorylation of I*κ*B proteins represents a convergence point for most signal transduction pathways under the oxidizing conditions leading to NF-*κ*B activation [36]. Recent data also disclosed that peroxynitrite stimulates NF-*κ*B activation via nitration of tyrosine in I*κ*B, thereby increasing I*κ*B degradation [37]. It has been speculated that posttranslational modification of transcription factors is a mechanism by which cells sense the redox changes [38]. Accordingly, PPAR*γ* nitration and subsequent inactivation may provide a signal for neurons sensing nitrosative condition in neurons.

Although HSYA is a hydrophilic drug with low oral bioavailability, the ability of HSYA to cross the blood-brain barrier has been confirmed previously following intravenous administration of HSYA [19]. Furthermore, since the blood-brain barrier is disrupted in varying degrees in numerous pathological conditions of the brain, like stroke or traumatic brain injury, it should not pose a significant obstacle to HSYA delivery in these settings. Indeed, evidence has proven that HSYA is absorbed in the brain tissues of the TBI rats after being orally administered with HSYA [12]. These data validated that the action of HSYA may result from its central effect in our study.

In our previous study, we have demonstrated that HSYA profoundly protected against tyrosine nitration elicited by authentic peroxynitrite in a cell-free system, indicating the role of HSYA as a peroxynitrite scavenger [2]. Accordingly, we speculated that HSYA may act through directly scavenging peroxynitrite and/or its derived radicals to inhibit nitro-PPAR*γ* formation in SIN-1-induced neurons. The structure of HSYA is quinochalcone c-glycoside [39]. Chalcones, a group of aromatic ketones, have been linked with antioxidant activity. Moreover, chalcones have been expected to directly react with peroxynitrite [40]. It is possible that HSYA could function as a competing substrate for peroxynitrite-triggered reaction and therefore protects free tyrosine or tyrosine residue of protein from nitrative modification. Whether HSYA specifically scavenges peroxynitrite, but not superoxide or NO, needs to be determined. Recently, HSYA was demonstrated to modulate endogenous antioxidant defenses of the brain by increasing the activity of antioxidant enzymes, including superoxide dismutase (SOD) and catalase (CAT), as well as the ratio of glutathione (GSH)/glutathione disulfide (GSSG) [12]. This effect may be also involved in its mechanism underlying the inhibition of PPAR*γ* nitration.

Our present data and others have shown that PPAR*γ* is crucial to the defensive mechanism of neurons against nitrosative stress and oxidative stress, both of which playing a role in the pathogenesis of many neurodegenerative diseases. Thus, keeping PPAR*γ* function active could become particularly important for the neurons in degenerative diseases. HSYA's protection against SIN-1-induced negative regulation of PPAR*γ* activity may help in potentiating the control of nitrosative stress and offer new therapeutic opportunities for treating neurodegenerative diseases.

## Data Availability

The data used to support the findings of this study are included within the article.
